# Dataset for the hybrid non-toughened and toughened epoxy adhesive properties

**DOI:** 10.1016/j.dib.2023.108912

**Published:** 2023-01-18

**Authors:** Dharun Vadugappatty Srinivasan, Anastasios P. Vassilopoulos

**Affiliations:** Composite Construction Laboratory (CCLab), Ecole Polytechnique Fédérale de Lausanne (EPFL), Station 16, Lausanne CH-1015, Switzerland

**Keywords:** Wind energy, Composites, Voids, Toughening, Epoxy adhesives, Micro-computed tomography, Digital image correlation, High speed imaging

## Abstract

In this article, the manufacturing and toughening effects on the material properties of epoxy adhesives used in wind turbine rotor blades are presented. Different adhesive materials are developed by combining SPABOND™ 820HTA (non-toughened) and SPABOND™ 840HTA (toughened) adhesives with the machine and manual mixing methods. Firstly, the manufacturing quality are compared between the two methods, in terms of void percentage and void volume using micro-computed tomography. Dynamical Mechanical analysis, uniaxial tensile testing, V-notch shear testing and single-edge-notch beam testing are carried out to evaluate the manufacturing and toughening effects. In these experiments, the digital image correlation technique is exploited to obtain the displacement and strain data. Origin Pro^Ⓡ^ and MATLAB R2021b^Ⓡ^ are utilized for technical data analysis, plotting, smoothing, filtering, and averaging. The obtained data could be used to select the adhesive material based on the strength and stiffness requirements, develop failure criteria, and predict the thick adhesive joint behavior by finite element modeling.

**Specifications Table**

**Table:** 

Subject	Materials science: Polymers and plastics
Specific subject area	Dynamic mechanical analysis, tensile, shear and fracture toughness properties of epoxy adhesives
Type of data	Table Figure
How the data were acquired	Different experiments were carried out and the corresponding data were acquired as following: • Micro computed tomography using Ultratom µCT system (RX solutions) • Dynamic mechanical analysis using TA^Ⓡ^ Q800 series equipment. • Uniaxial tensile testing using MTS^Ⓡ^ 810 Landmark servo-hydraulic machine with calibrated load cell of 5 kN. • V-notch shear testing Walter + bai (w + b) test machine with local of 50 kN. • Single-edge-notch bending test using MTS^Ⓡ^ Acumen equipped with 3 kN load cell. • Imaging for digital image correlation (DIC) using Point Grey – Grasshopper 3 camera (2.2 Megapixels) housing Fujinon HF35SA-1 35 mm F/1.4 lens. • Digital image correlation using VIC 2D-6 software from correlated solutions^Ⓡ^ • An inhouse developed LabVIEW^Ⓡ^ software for acquiring images and forces values from the test machine. • Sony XCG-5005E (5 Megapixels) camera with 2448 × 2048 pixels resolution. • Origin Pro^Ⓡ^ software for smoothing, filtering, and averaging the plots.
Data format	Raw Analyzed Filtered
Description of data collection	Avizo^Ⓡ^ software was used to reconstruct the micro-computed tomography images and analyse the void parameters of all adhesive materials. The storage modulus, tan delta and the glass transition temperature were identified by TA universal analysis^Ⓡ^. The corresponding plots were plotted with Origin Pro^Ⓡ^ software. The average curves of multiple specimens with standard deviation were also realised with Origin Pro^Ⓡ^ software. The engineering stress and strain values were obtained through DIC analysis. From these values, the true stress and strain were calculated and smoothened using Origin Pro^Ⓡ^ software. The tensile properties such as Youngs modulus, 0.2% yield strength, ultimate strength, failure strain and tensile toughness were calculated by MATLAB R2021b^Ⓡ^ software program. The same procedure was followed for v-notch shear test to plot the shear stress versus shear strain curves. In SENB tests, the force and displacement values were calculated through DIC analysis and the fracture toughness versus displacement plot values were derived by a MATLAB R2021b^Ⓡ^ software program. Overall, Origin Pro^Ⓡ^ software was used for smoothing, filtering, and averaging the plots.
Data source location	The Structural Engineering Platform, GIS-ENAC (https://www.epfl.ch/schools/enac/research/platforms-and-services/gis/), Composite Construction Laboratory (CCLab)/ Ecole Polytechnique Fédérale de Lausanne (EPFL), Lausanne, Switzerland.
Data accessibility	Repository name: Mendeley Data Data identification number: https://doi.org/10.17632/5rgwzw6jn3.1 Direct URL to data: https://data.mendeley.com/datasets/5rgwzw6jn3/1
Related research article	D.V. Srinivasan, A.P. Vassilopoulos, Manufacturing and toughening effects on the material properties of wind turbine blade adhesives, Polym. Test. 116 (2022) 107770. https://doi.org/10.1016/J.POLYMERTESTING.2022.107770 .

## Value of the Data

• The effect of machine and manual-mixed manufacturing methods on the adhesive material properties are quantified with the presented data.
• The adhesive toughening effect on the material properties is determined. Also, the relationship between tensile and shear properties is deducted.
• An appropriate adhesive failure criterion can be developed with the available data.
• Data could be used to predict the thick adhesive joint/structure behavior using suitable finite element models.

## 1 Data Description

This article describes the raw, processed and analyzed data on the effect of manufacturing and toughening effect on the material properties of wind turbine blade adhesives. Herein, the data collection process and experimental data of each specimen of different adhesive materials groups are presented whereas the conclusive results are found in [1]. The plots (Fig. 1, Fig. 2, Fig. 3, Fig. 4, Fig. 5, Fig. 6, Fig. 7, Fig. 8, Fig. 9, Fig. 10, Fig. 11, Fig. 12, Fig. 13, Fig. 14, Fig. 15, Fig. 16, Fig. 17, Fig. 18, Fig. 19, Fig. 20, Fig. 21) can be replicated using the published Mendeley data [2]. Table 1 provides the figure and table captions and their associated data file (Tables 2–28).

**Table 1:** Guidelines for referring the data files.

S.No	Table/Figure no	Mendeley data file	Comments
1	Table 2	Micro-CT.xlsx	-
2	Fig. 1, Fig. 2, Fig. 3, Fig. 4, Fig. 5, Fig. 6, Fig. 7, Fig. 8	DMA.xlsx	Each adhesive data provided in individual sheets
3	Fig. 10, Fig. 11, Fig. 12, Fig. 13, Fig. 14, Fig. 15, Fig. 16, Fig. 17	Tensile.xlsx	Each adhesive data provided in individual sheets
4	Figs. 18 and 19	Shear.xlsx	Each adhesive data provided in individual sheets
5	Figs. 20 and 21	SENB.xlsx	Each adhesive data provided in individual sheets

### 1.1 Micro-computed tomography (µCT) scanning data

**Table 2:** Void analysis data of the pristine and hybrid adhesives.

Adhesive	Total volume *(*$mm^{3}$*)*	Void volume *(*$mm^{3}$*)*	Void percentage (%)	Void quantity
BBM1	81.241	0.084	0.104	4511
BTM1	85.124	0.008	0.009	2168
TBM1	78.061	0.032	0.040	430
TTM1	88.589	0.232	0.262	133
BBM2	70.316	0.183	0.260	95
BTM2	66.133	2.475	3.742	4636
TBM2	71.219	1.815	2.548	2136
TTM2	66.115	0.008	0.012	13547

### 1.2 Dynamic mechanical analysis data

**Fig. 1:**
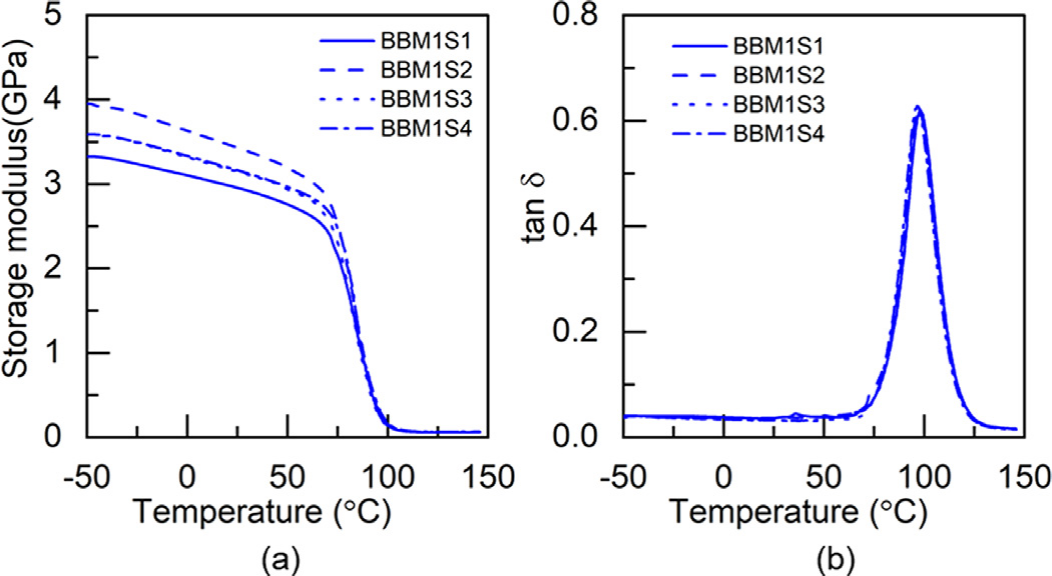
DMA test results of BBM1 adhesive: (a) storage modulus and (b) tan δ.

**Fig. 2:**
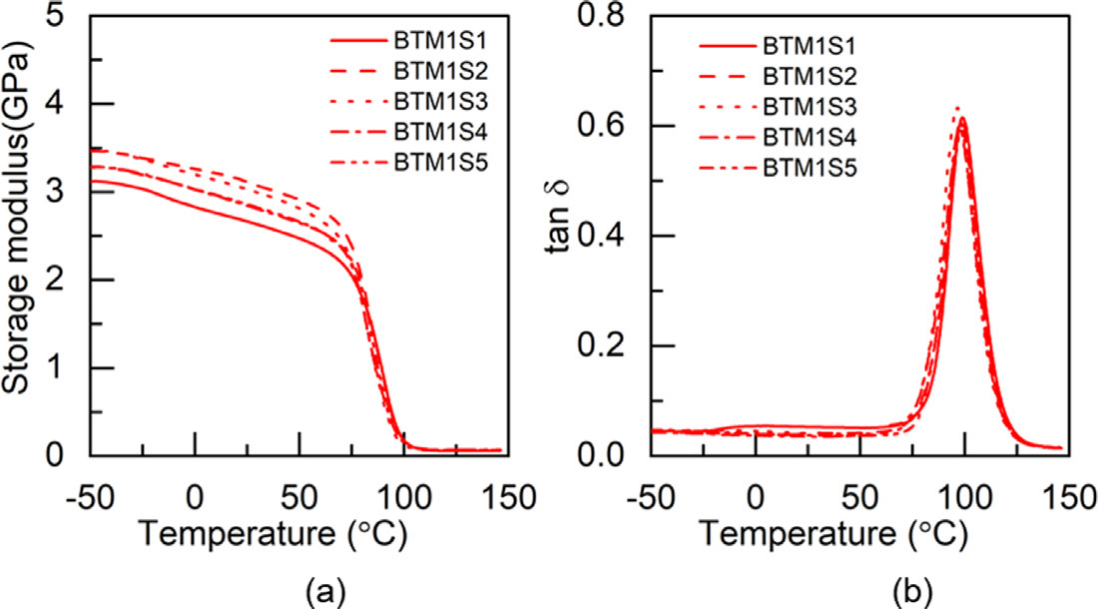
DMA test results of BTM1 adhesive: (a) storage modulus and (b) tan δ.

**Fig. 3:**
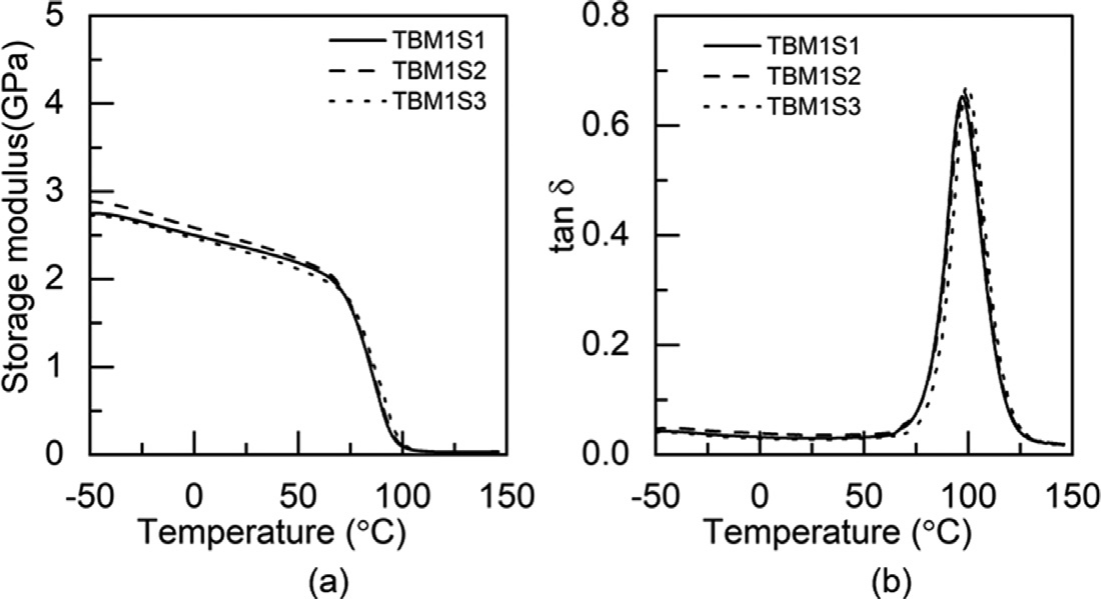
DMA test results of TBM1 adhesive: (a) storage modulus and (b) tan δ.

**Fig. 4:**
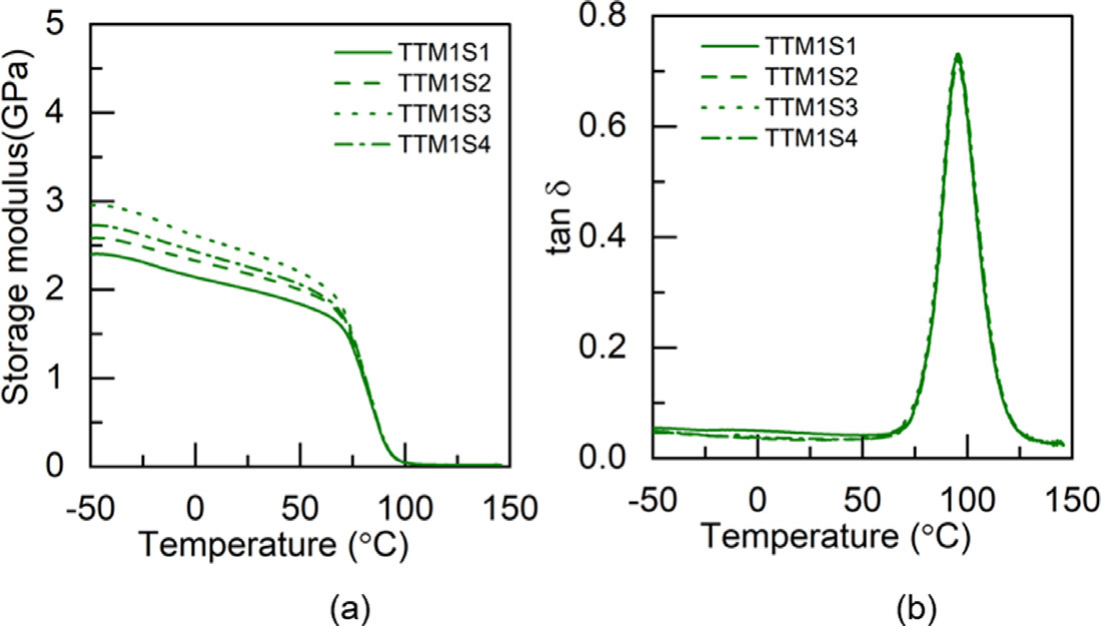
DMA test results of TTM1 adhesive: (a) storage modulus and (b) tan δ.

**Fig. 5:**
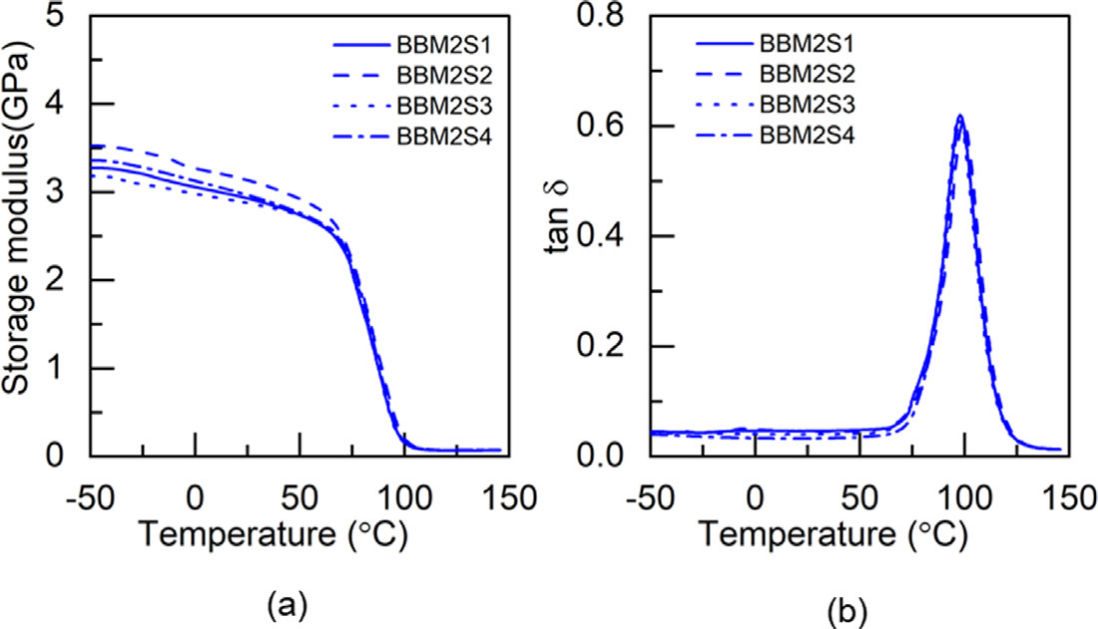
DMA test results of BBM2 adhesive: (a) storage modulus and (b) tanδ.

**Fig. 6:**
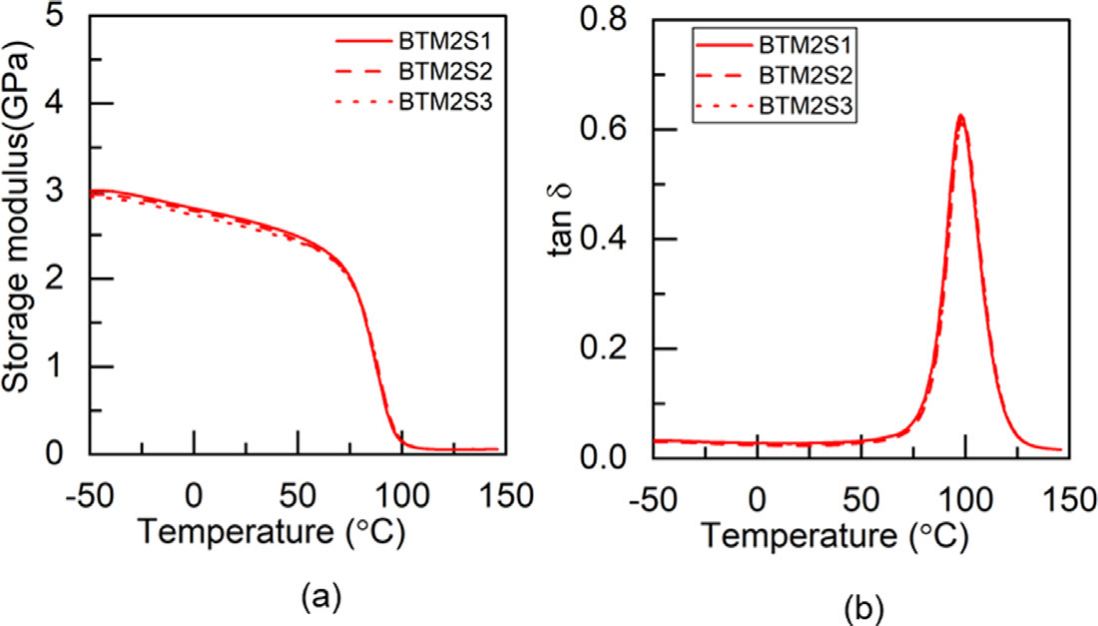
DMA test results of BTM2 adhesive: (a) storage modulus and (b) tan δ.

**Fig. 7:**
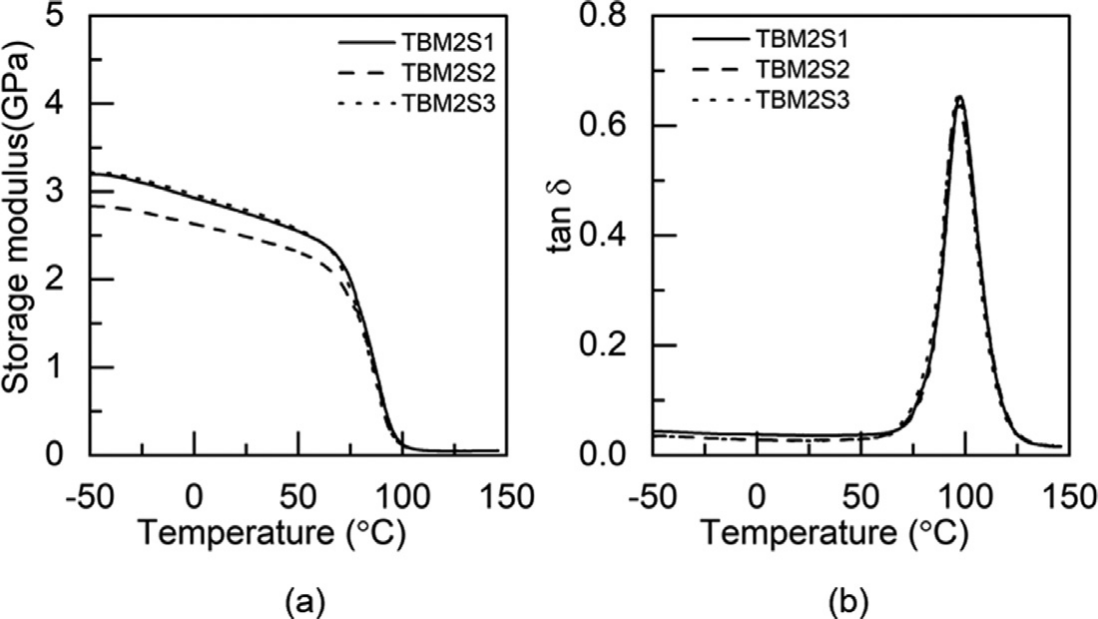
DMA test results of TBM2 adhesive: (a) storage modulus and (b) tan δ

**Fig. 8:**
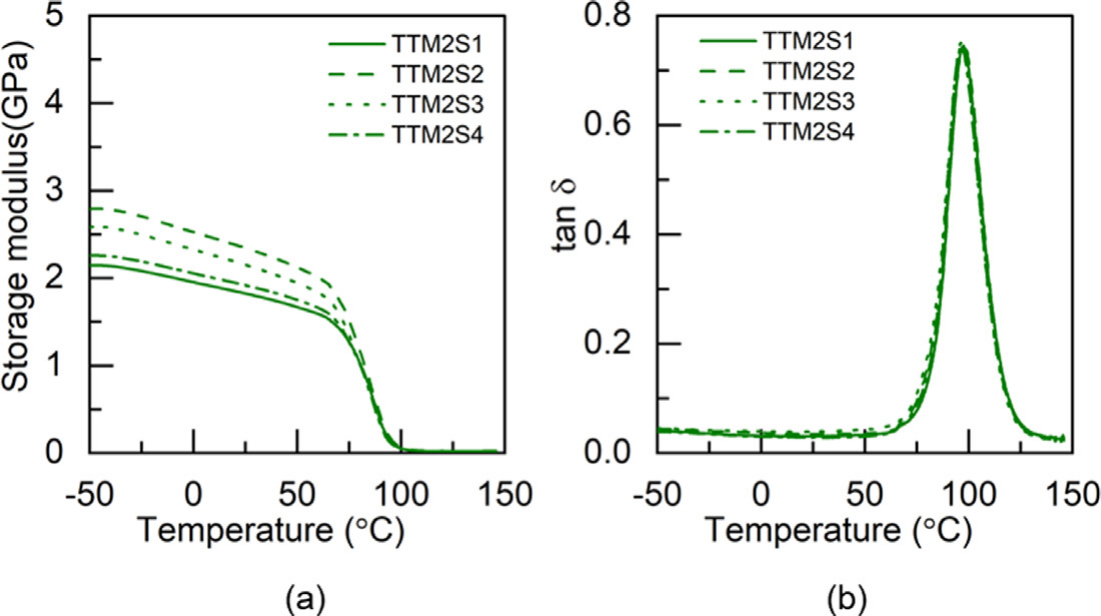
DMA test results of TTM2 adhesive: (a) storage modulus and (b) tan δ.

**Fig. 9:**
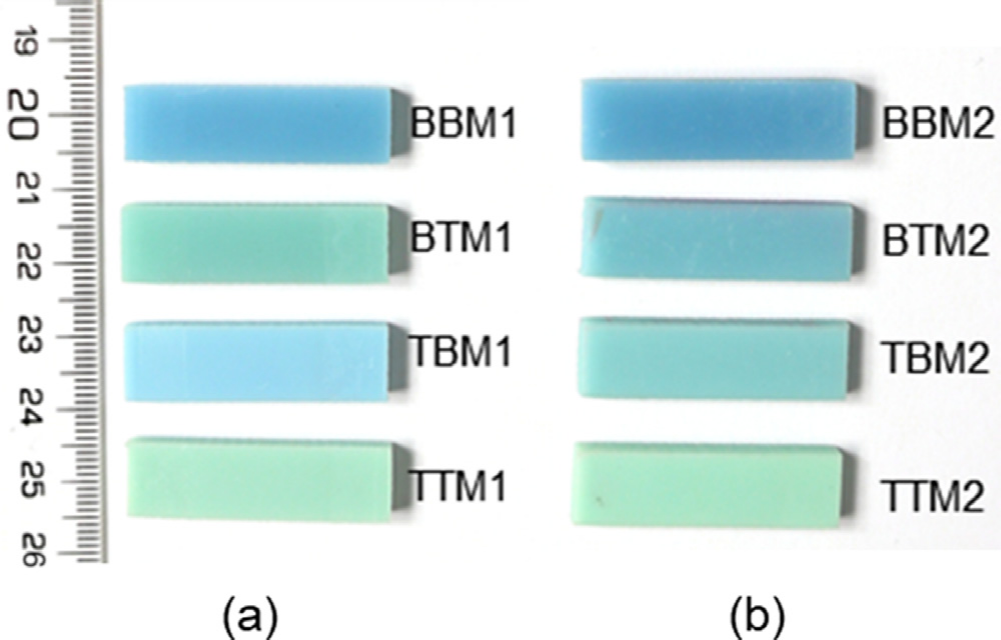
DMA tested specimens: (a) M1 adhesives and (b) M2 adhesives.

**Table 3:** Glass transition temperature of the pristine and hybrid adhesives fabricated through M1 method.

	Glass transition temperature (°C)					
Specimen	S1	S2	S3	S4	S5	Average
BBM1	73.4	74.1	73.4	72.8	-	73.4±0.5
BTM1	78.4	74.4	73.3	74.4	76.5	75.4±1.8
TBM1	73.3	73.5	76.7	-	-	74.5±1.6
TTM1	71.9	70.9	70.6	72.2	-	71.4±0.7

**Table 4:** Glass transition temperature of the pristine and hybrid adhesives fabricated through M2 method.

	Glass transition temperature (°C)					
Specimen	S1	S2	S3	S4	S5	Average
BBM2	71.9	71.8	72.8	73.8	-	72.6±0.8
BTM2	76.4	77.4	76.9	-	-	76.9±0.4
TBM2	78.8	77.6	72.0	-	-	76.1±2.9
TTM2	75.1	71.1	71.2	73.9	-	72.8±1.7

### 1.3 Uniaxial tensile data

**Fig. 10:**
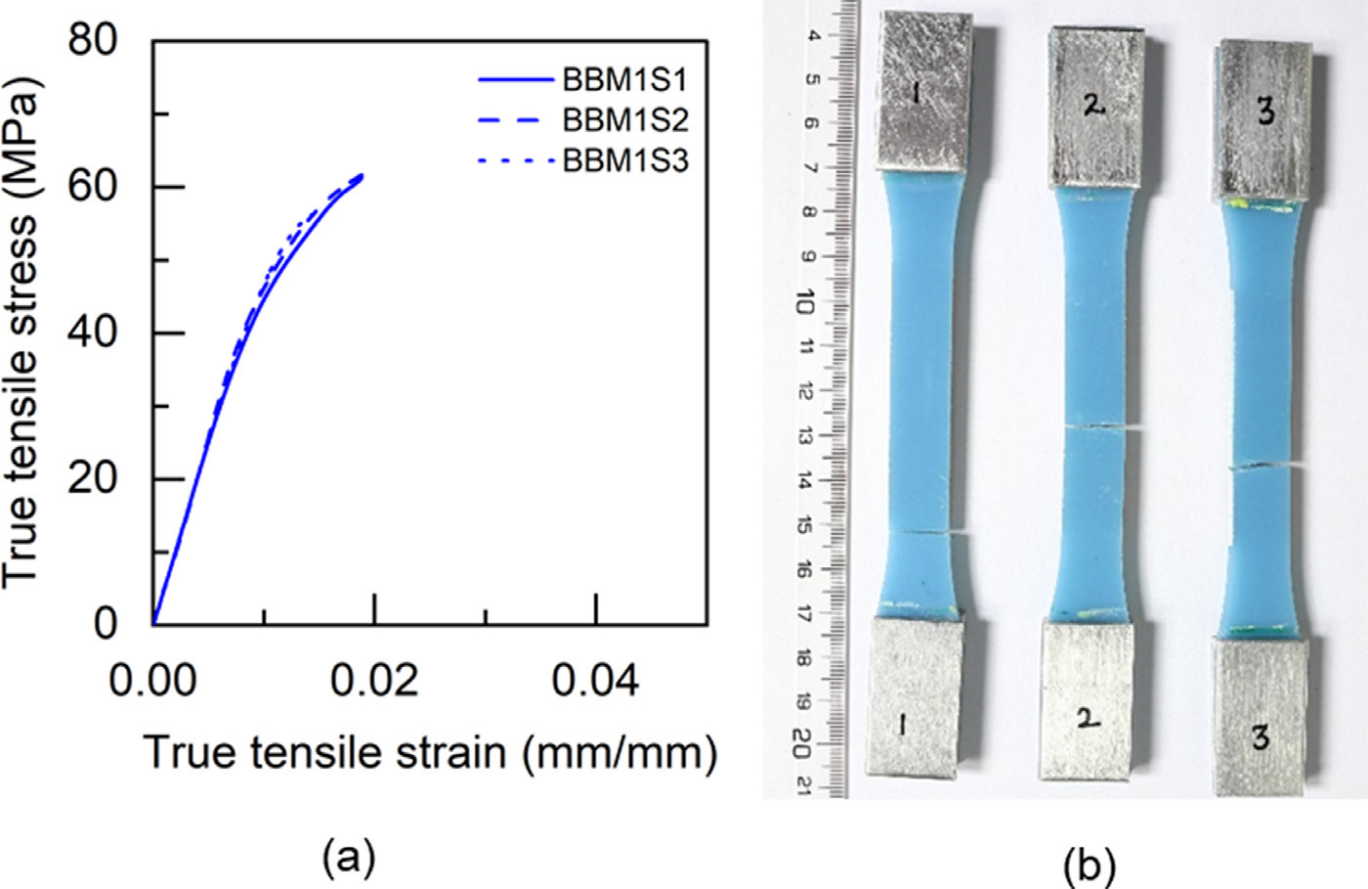
BBM1 adhesive: (a) tensile stress versus tensile strain and (b) after tensile failure.

**Fig. 11:**
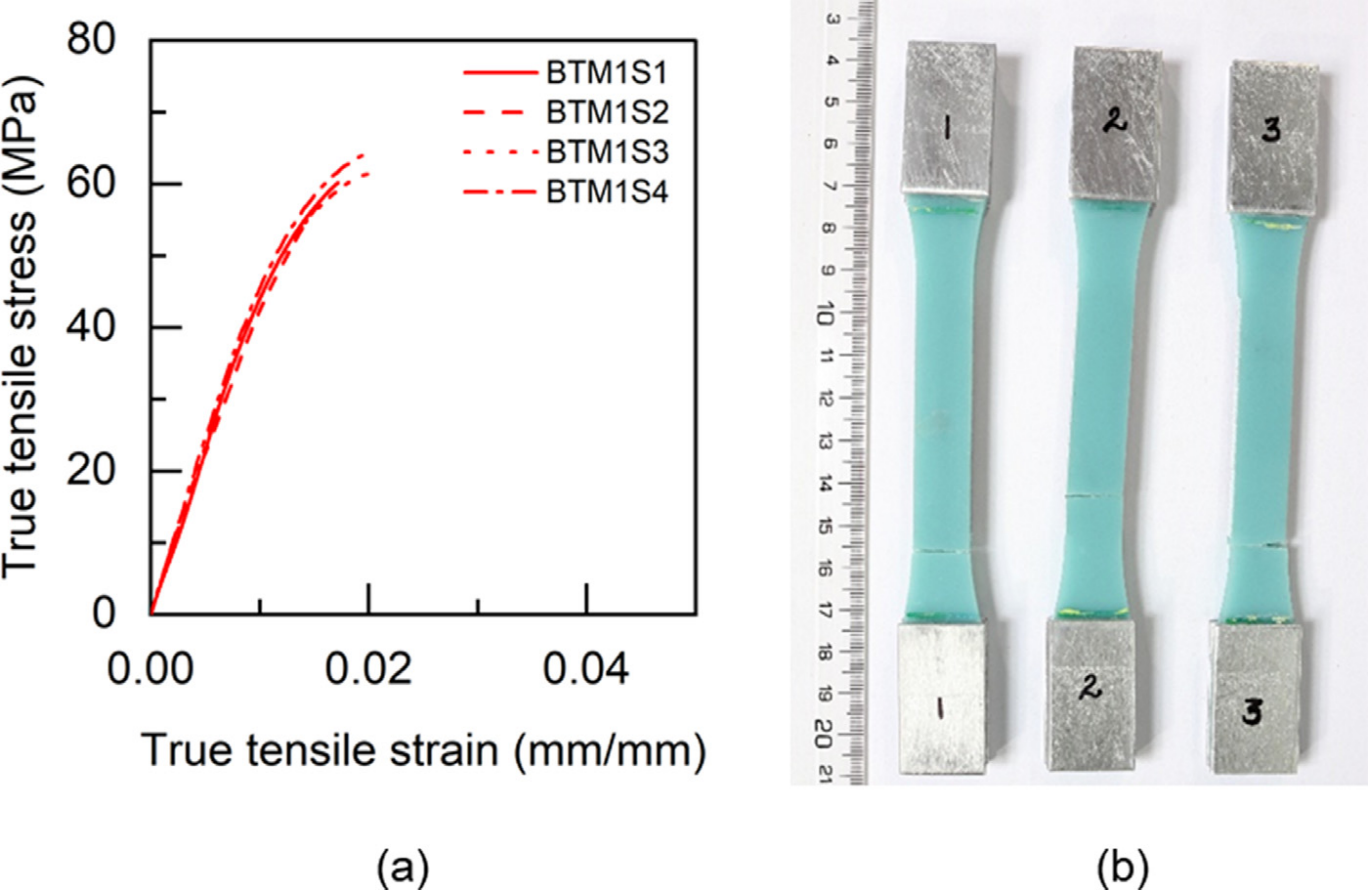
BTM1 adhesive: (a) tensile stress versus tensile strain and (b) after tensile failure.

**Fig. 12:**
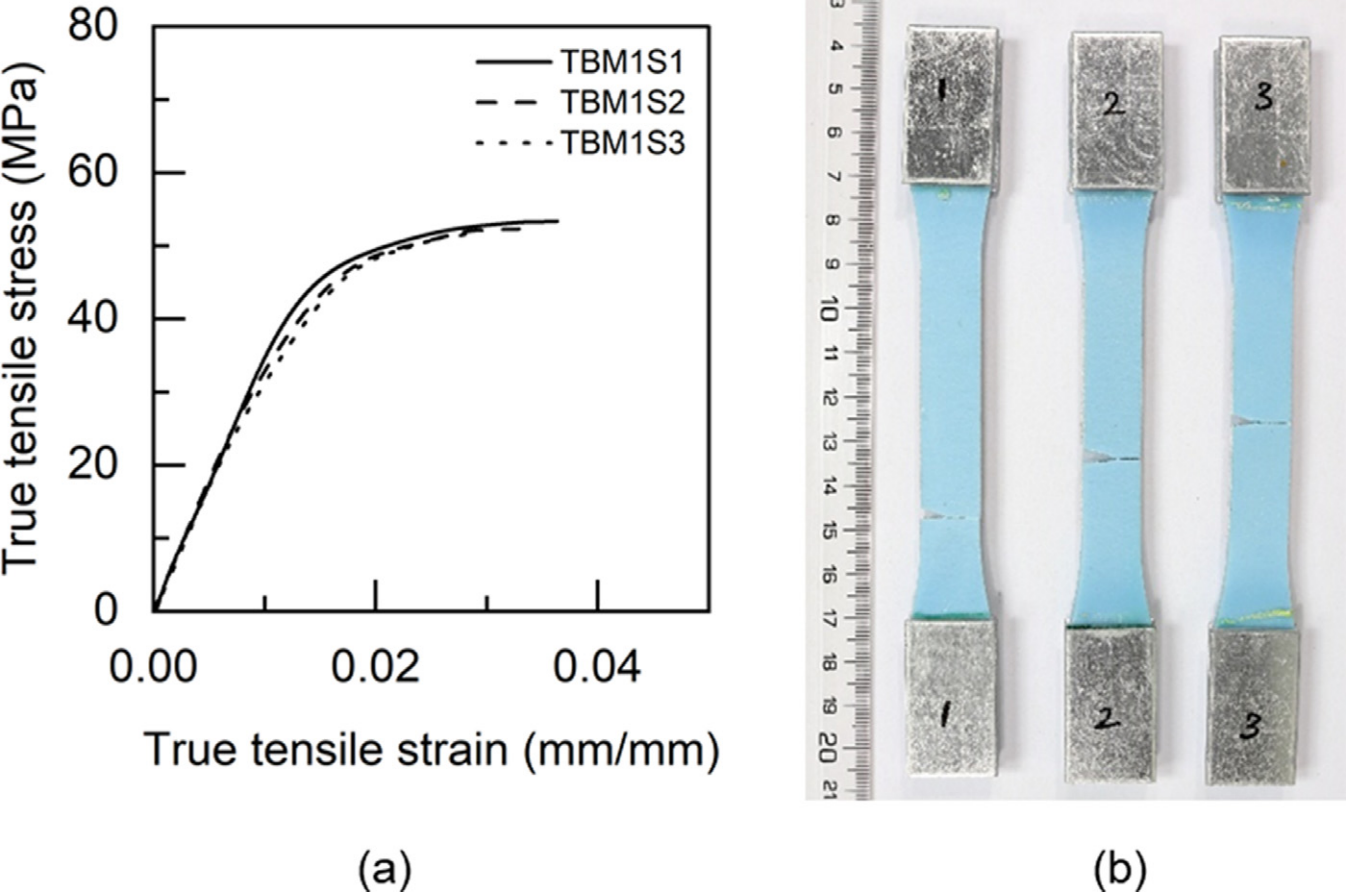
TBM1 adhesive: (a) tensile stress versus tensile strain and (b) after tensile failure.

**Fig. 13:**
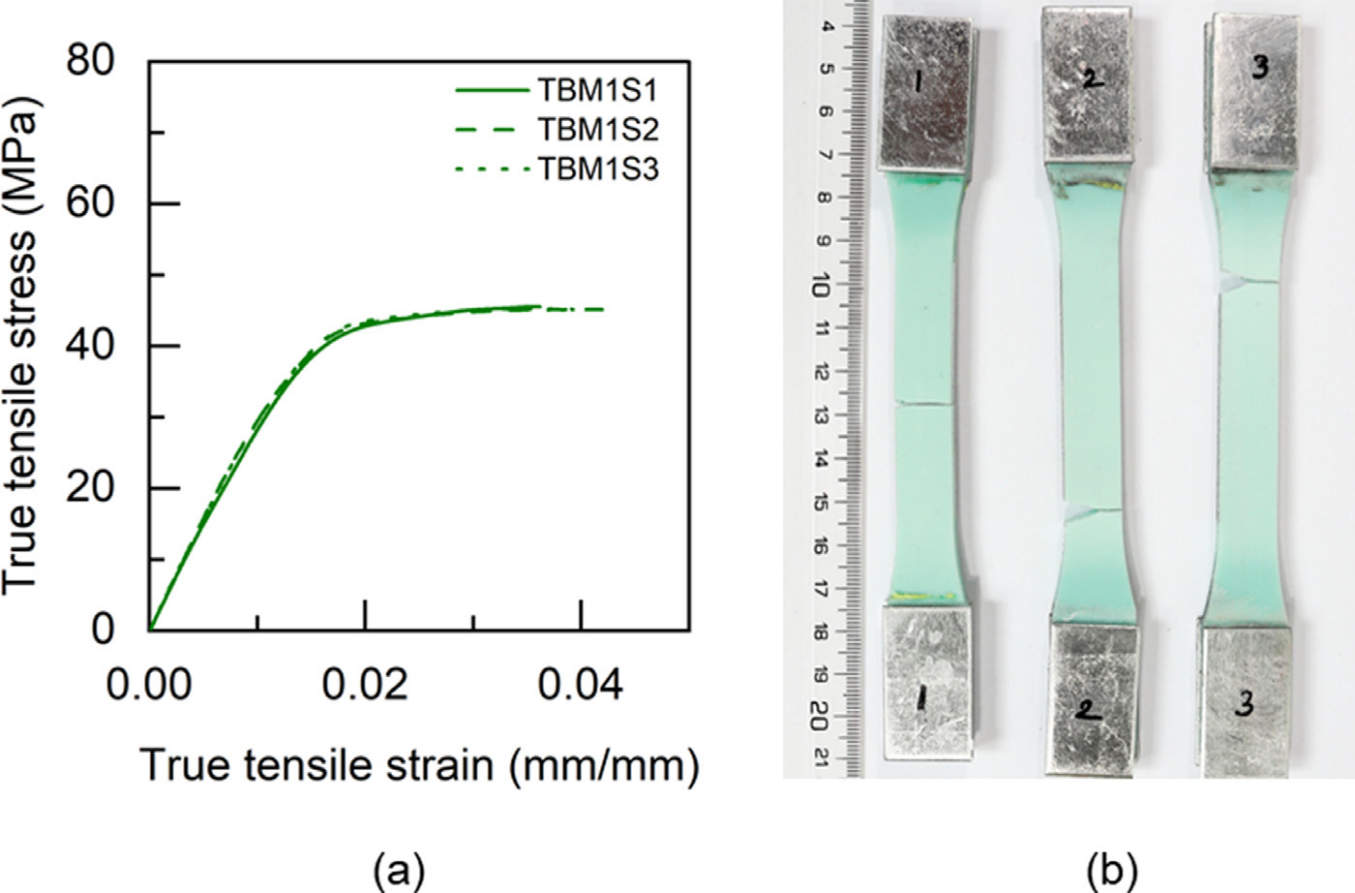
TTM1 adhesive: (a) tensile stress versus tensile strain and (b) after tensile failure.

**Fig. 14:**
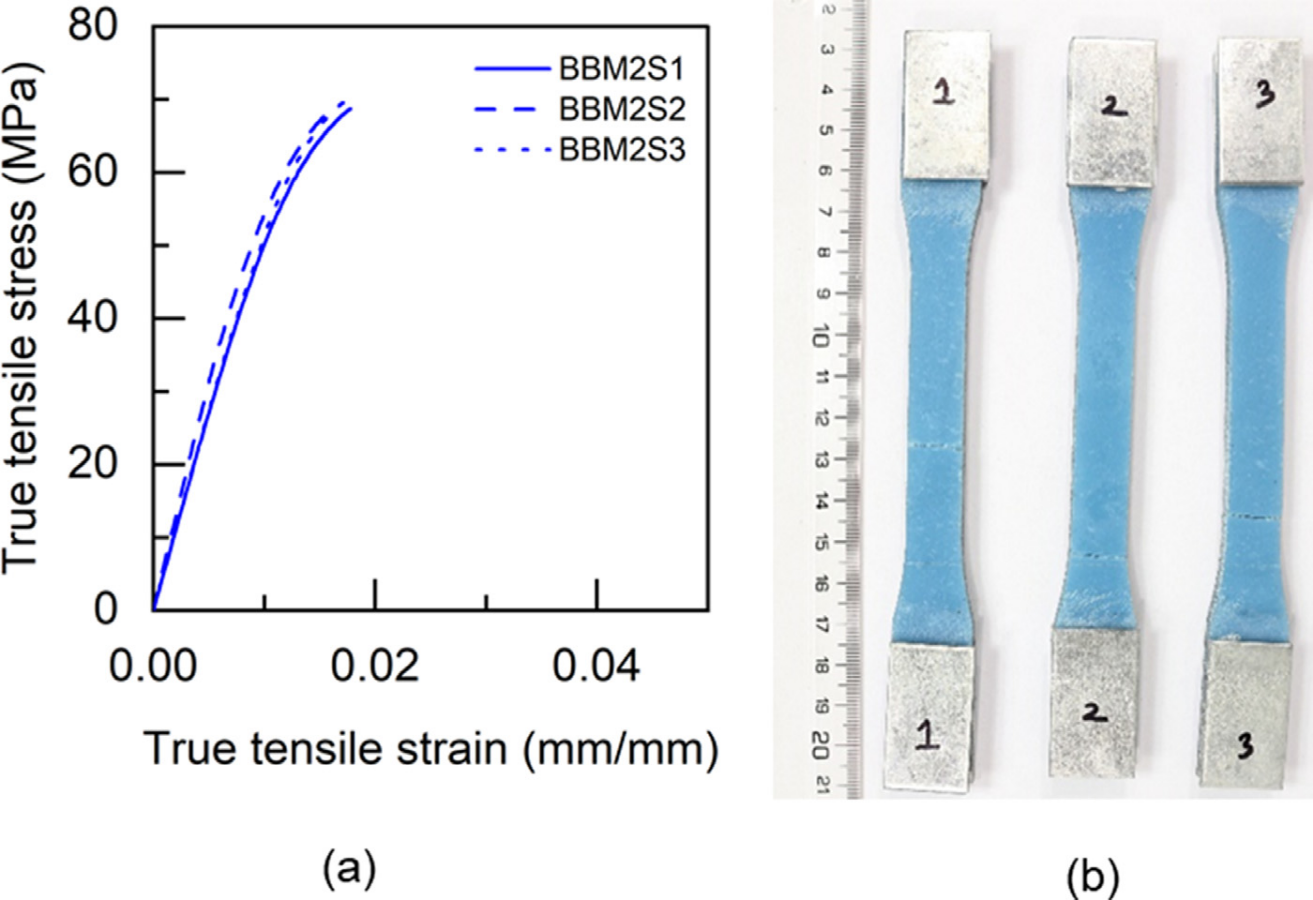
BBM2 adhesive: (a) tensile stress versus tensile strain and (b) after tensile failure.

**Fig. 15:**
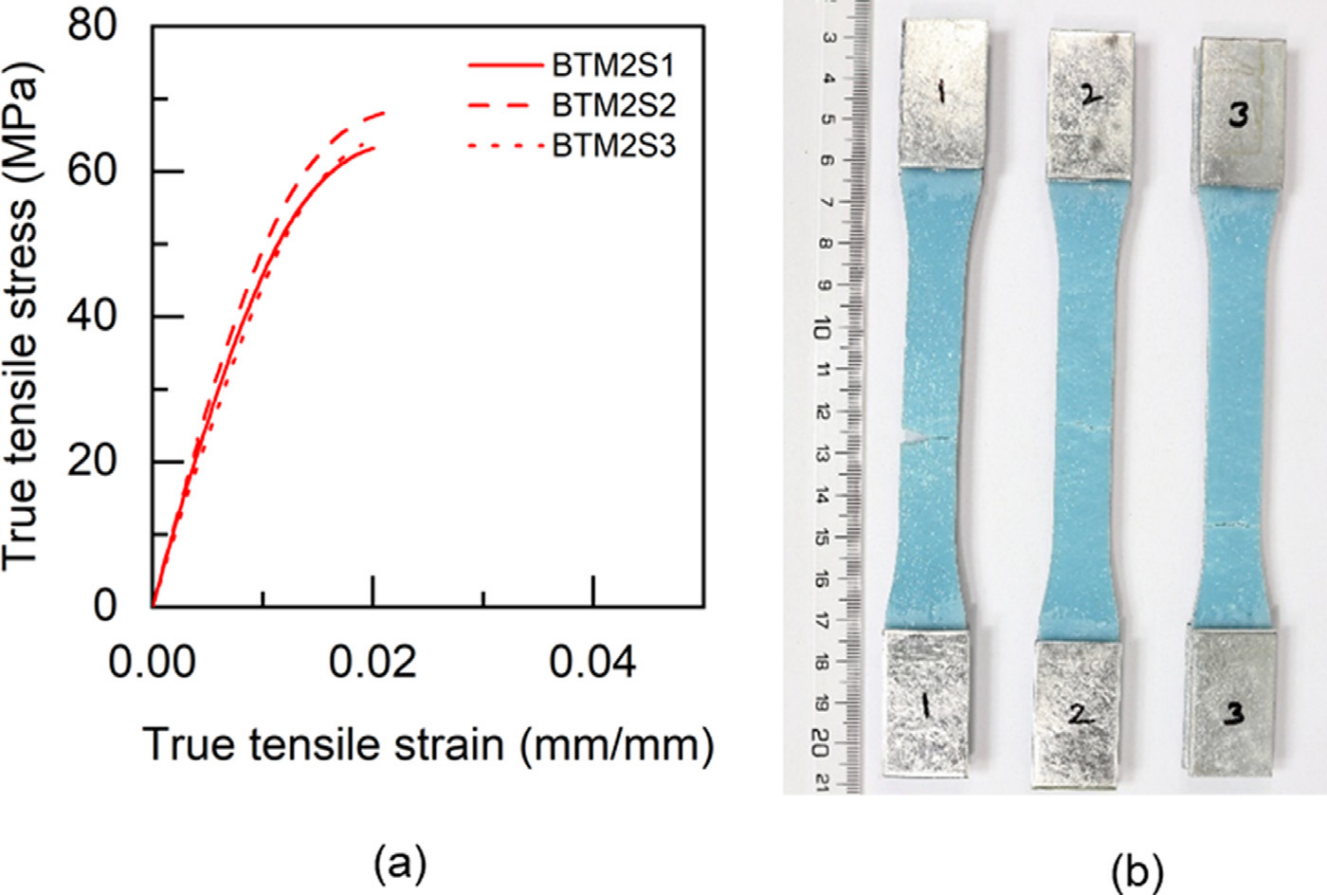
BTM2 adhesive: (a) tensile stress versus tensile strain and (b) after tensile failure.

**Fig. 16:**
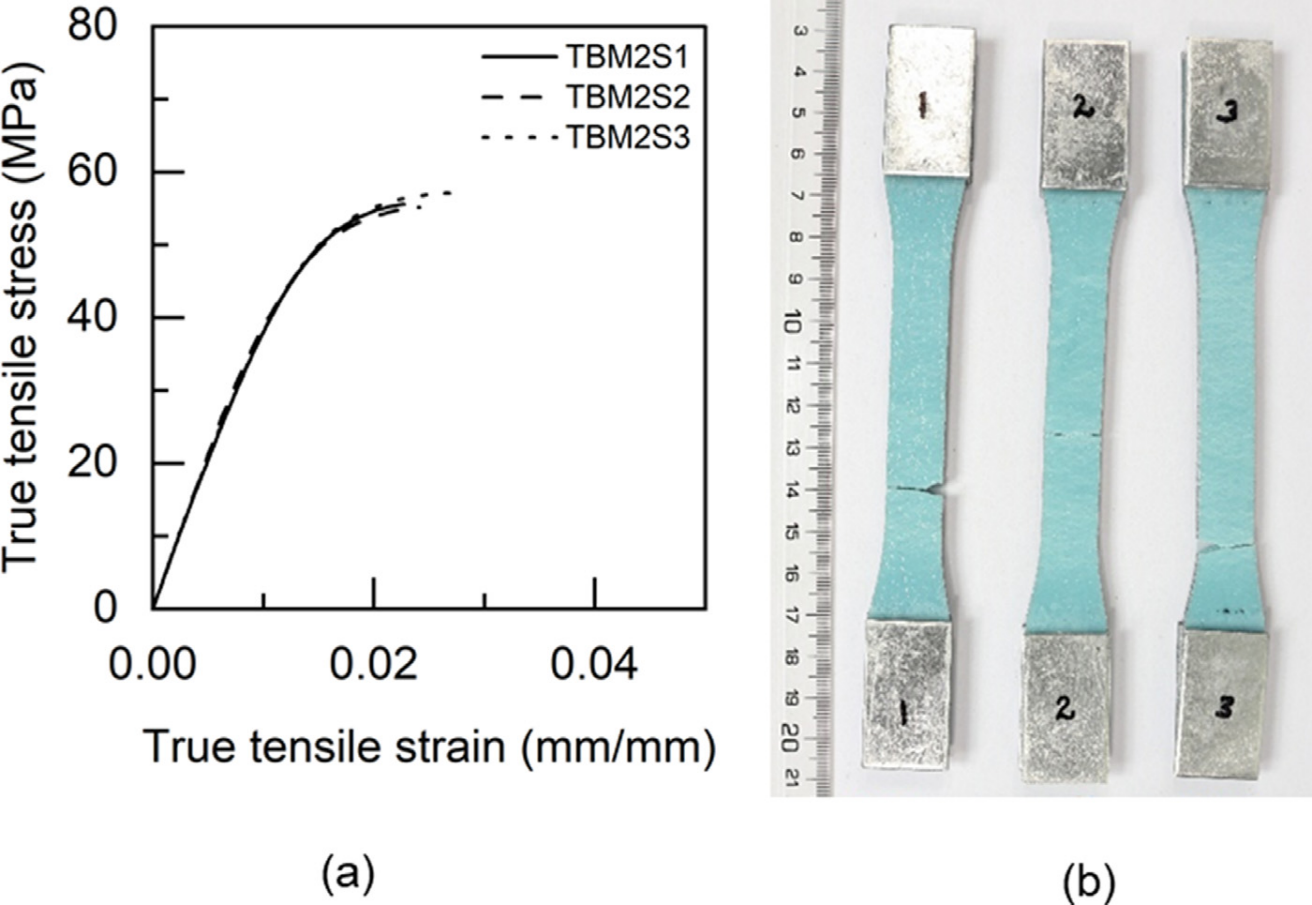
TBM2 adhesive: (a) tensile stress versus tensile strain and (b) after tensile failure.

**Fig. 17:**
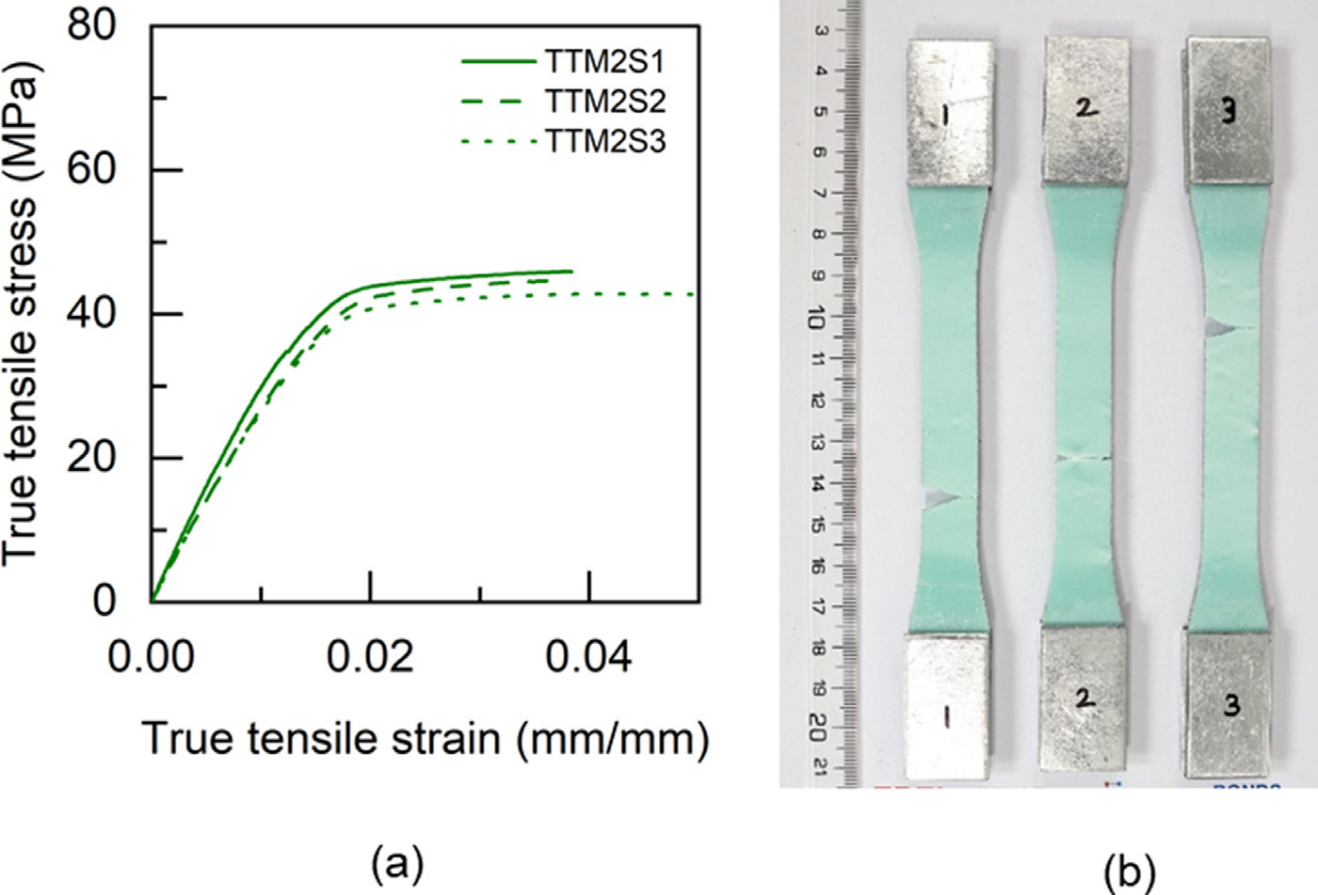
TTM2 adhesive: (a) tensile stress versus tensile strain and (b) after tensile failure.

**Table 5:** Uniaxial tensile properties of BBM1 adhesive.

		BBM1			
Property	Unit	S1	S2	S3	Average
Young's modulus (E)	GPa	5.01	5.2	5.09	5.1±0.08
0.2% offset Yield stress $\left(\sigma_{y}\right)$	MPa	49.21	50.97	53.8	51.38±1.89
0.2% offset Yield strain $\left(\varepsilon_{y}\right)$	mm/mm	0.0118	0.0119	0.0126	0.0121±0.0003
Tensile toughness $\left(U_{T}\right)$	$kJ/m^{3}$	0.72	0.82	0.49	0.68±0.14
Maximum stress $\left(\sigma_{u}\right)$	MPa	61.34	62.65	56.48	60.16±2.65
Strain at failure $\left(\varepsilon_{f}\right)$	mm/mm	0.0188	0.0203	0.0147	0.0179±0.0024

**Table 6:** Uniaxial tensile properties of BTM1 adhesive.

		BTM1				
Property	Unit	S1	S2	S3	S4	Average
Young's modulus (E)	GPa	4.63	4.23	4.6	4.83	4.57±0.22
0.2% offset Yield stress $\left(\sigma_{y}\right)$	MPa	54.47	56.51	53.39	54.75	54.78±1.12
0.2% offset Yield strain $\left(\varepsilon_{y}\right)$	mm/mm	0.0138	0.0151	0.0135	0.0133	0.0139±0.0007
Tensile toughness $\left(U_{T}\right)$	$kJ/m^{3}$	0.6083	0.5493	0.8051	0.8079	0.69±0.12
Maximum stress $\left(\sigma_{u}\right)$	MPa	60.17	58.52	61.5	64.47	61.17±2.18
Strain at failure $\left(\varepsilon_{f}\right)$	mm/mm	0.0172	0.0163	0.0204	0.0199	0.0184±0.0017

**Table 7:** Uniaxial tensile properties of TBM1 adhesive.

		TBM1			
Property	Unit	S1	S2	S3	Average
Young's modulus (E)	GPa	3.47	3.5	3.33	3.43±0.07
0.2% offset Yield stress $\left(\sigma_{y}\right)$	MPa	45.40	40.76	40.44	42.20±2.27
0.2% offset Yield strain $\left(\varepsilon_{y}\right)$	mm/mm	0.015	0.0136	0.0141	0.0142±0.0006
Tensile toughness $\left(U_{T}\right)$	$kJ/m^{3}$	1.47	1.26	1.06	1.26±0.17
Maximum stress $\left(\sigma_{u}\right)$	MPa	53.42	52.35	51.9	52.56±0.64
Strain at failure $\left(\varepsilon_{f}\right)$	mm/mm	0.0363	0.0329	0.0296	0.0329±0.0027

**Table 8:** Uniaxial tensile properties of TTM1 adhesive.

		TTM1			
Property	Unit	S1	S2	S3	Average
Young's modulus (E)	GPa	2.79	3.11	3.05	2.98±0.14
0.2% offset Yield stress $\left(\sigma_{y}\right)$	MPa	39.86	37.72	38.48	38.69±0.89
0.2% offset Yield strain $\left(\varepsilon_{y}\right)$	mm/mm	0.0161	0.0141	0.0145	0.0149±0.0009
Tensile toughness $\left(U_{T}\right)$	$kJ/m^{3}$	1.24	1.51	1.40	1.38±0.11
Maximum stress $\left(\sigma_{u}\right)$	MPa	45.56	45.21	45.3	45.36±0.15
Strain at failure $\left(\varepsilon_{f}\right)$	mm/mm	0.0362	0.0419	0.0392	0.0391±0.0023

**Table 9:** Uniaxial tensile properties of BBM2 adhesive.

		BBM2			
Property	Unit	S1	S2	S3	Average
Young's modulus (E)	GPa	5.26	6.14	5.37	5.59±0.39
0.2% offset Yield stress $\left(\sigma_{y}\right)$	MPa	61.38	59.37	63.65	61.47±1.75
0.2% offset Yield strain $\left(\varepsilon_{y}\right)$	mm/mm	0.0136	0.0116	0.0138	0.0130±0.0010
Tensile toughness $\left(U_{T}\right)$	$kJ/m^{3}$	0.74	0.67	0.71	0.71±0.03
Maximum stress $\left(\sigma_{u}\right)$	MPa	68.74	68.57	69.73	69.01±0.51
Strain at failure $\left(\varepsilon_{f}\right)$	mm/mm	0.0178	0.016	0.0171	0.0170±0.0007

**Table 10:** Uniaxial tensile properties of BTM2 adhesive.

		BTM2			
Property	Unit	S1	S2	S3	Average
Young's modulus (E)	GPa	4.99	5.33	4.54	4.95±0.32
0.2% offset Yield stress $\left(\sigma_{y}\right)$	MPa	52.9	57.01	57.05	55.65±1.95
0.2% offset Yield strain $\left(\varepsilon_{y}\right)$	mm/mm	0.0125	0.0126	0.0144	0.0132±0.0009
Tensile toughness $\left(U_{T}\right)$	$\text{kJ}/m^{3}$	0.82	0.97	0.74	0.84±0.10
Maximum stress $\left(\sigma_{u}\right)$	MPa	63.21	68.15	63.68	65.01±2.23
Strain at failure $\left(\varepsilon_{f}\right)$	mm/mm	0.02	0.0214	0.019	0.0201±0.0010

**Table 11:** Uniaxial tensile properties of TBM2 adhesive.

		TBM2			
Property	Unit	S1	S2	S3	Average
Young's modulus (E)	GPa	3.97	4	4.1	4.02±0.06
0.2% offset Yield stress $\left(\sigma_{y}\right)$	MPa	47.68	47.22	46.73	47.21±0.39
0.2% offset Yield strain $\left(\varepsilon_{y}\right)$	mm/mm	0.0139	0.0136	0.0133	0.0136±0.0002
Tensile toughness $\left(U_{T}\right)$	$kJ/m^{3}$	0.84	0.92	1.11	0.96±0.11
Maximum stress $\left(\sigma_{u}\right)$	MPa	55.62	55.15	57.18	55.98±0.87
Strain at failure $\left(\varepsilon_{f}\right)$	mm/mm	0.0227	0.0241	0.0275	0.0248±0.0020

**Table 12:** Uniaxial tensile properties of TTM2 adhesive.

		TTM2			
Property	Unit	S1	S2	S3	Average
Young's modulus (E)	GPa	3.03	2.71	2.68	2.81±0.16
0.2% offset Yield stress $\left(\sigma_{y}\right)$	MPa	38.27	38.22	37.08	37.86±0.55
0.2% offset Yield strain $\left(\varepsilon_{y}\right)$	mm/mm	0.0144	0.0159	0.0157	0.0153±0.0007
Tensile toughness $\left(U_{T}\right)$	$kJ/m^{3}$	1.37	1.26	1.73	1.45±0.20
Maximum stress $\left(\sigma_{u}\right)$	MPa	45.92	44.63	42.85	44.47±1.26
Strain at failure $\left(\varepsilon_{f}\right)$	mm/mm	0.0384	0.0373	0.0493	0.0417±0.0054

### 1.4 V-notch shear data

**Fig. 18:**
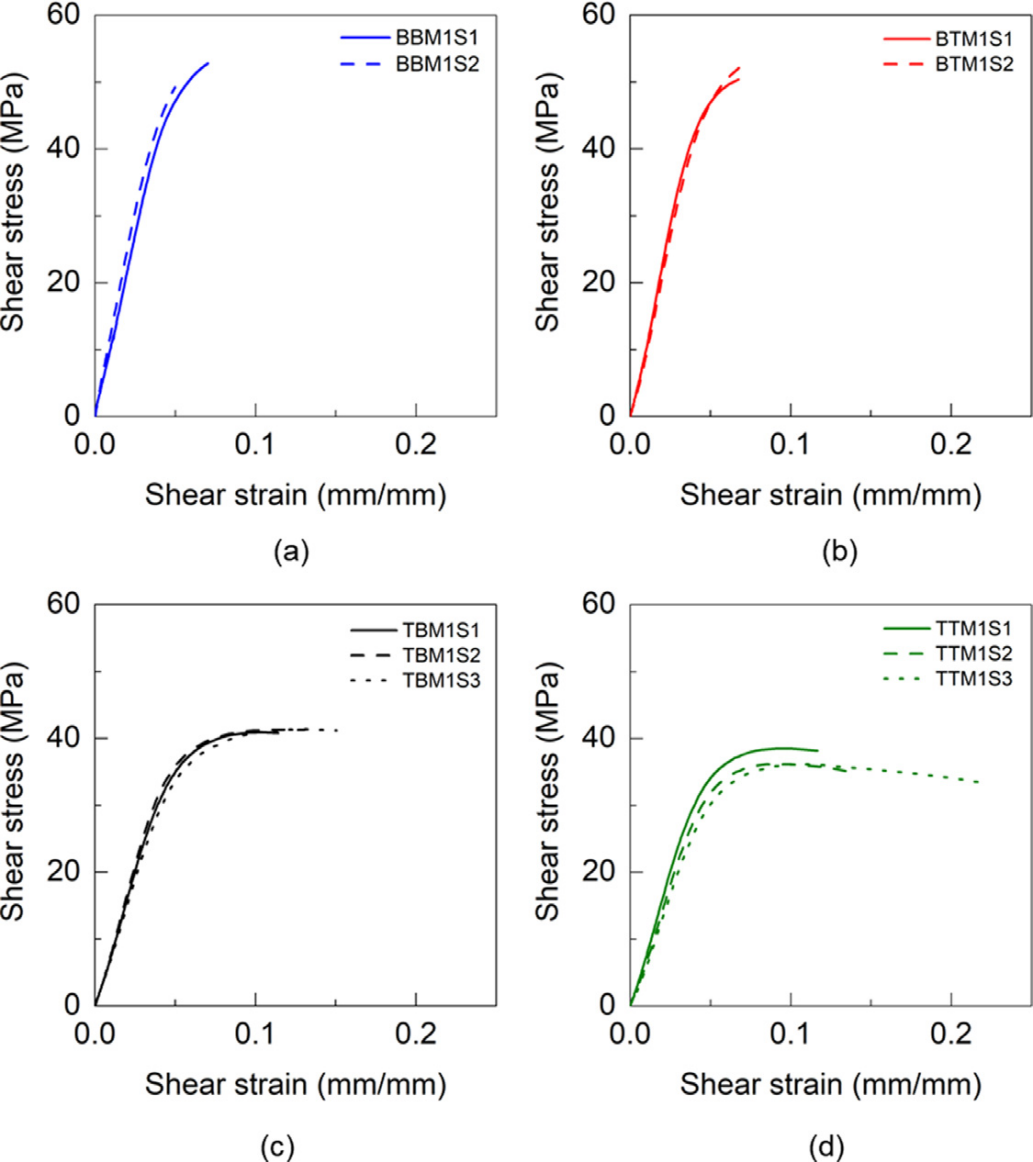
Shear stress and shear strain response of adhesives (a) BBM1, BTM1, TBM1 and TTM1.

**Fig. 19:**
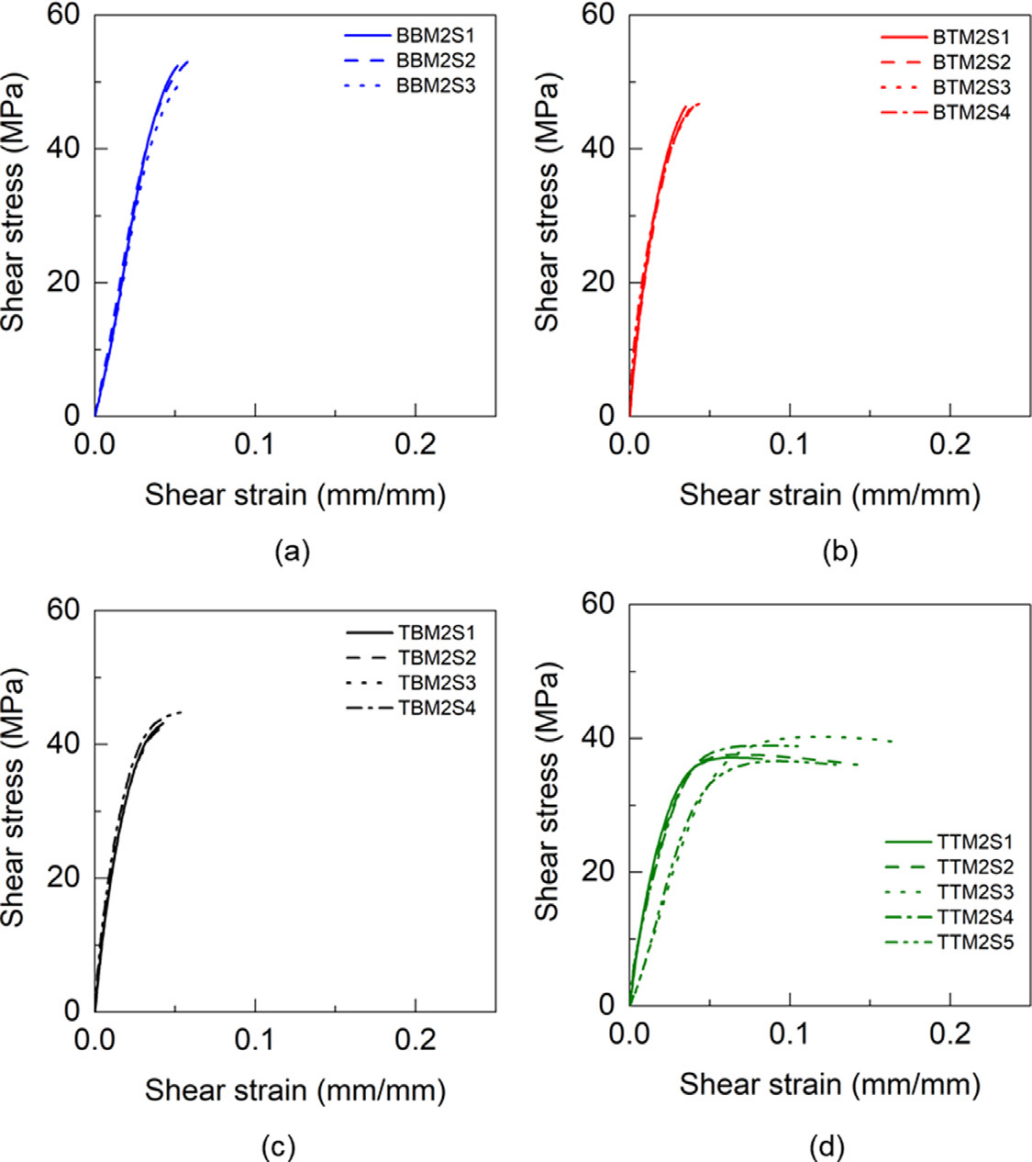
Shear stress and shear strain response of BTM2 adhesive.

**Table 13:** Shear properties of BBM1 adhesive.

		BBM1		
Property	Unit	S1	S2	Average
Shear modulus (G)	GPa	1.07	1.24	1.16±0.09
Maximum shear stress $\left(\tau_{u}\right)$	MPa	52.8	49.23	51.02±1.79
Shear strain at failure $\left(\gamma_{f}\right)$	mm/mm	0.0703	0.0499	0.0601±0.0102

**Table 14:** Shear properties of BTM1 adhesive.

		BTM1		
Property	Unit	S1	S2	Average
Shear modulus (G)	GPa	1.16	1.09	1.13±0.03
Maximum shear stress $\left(\tau_{u}\right)$	MPa	50.36	52.64	51.50±1.14
Shear strain at failure $\left(\gamma_{f}\right)$	mm/mm	0.0676	0.0714	0.0695±0.0019

**Table 15:** Shear properties of TBM1 adhesive.

		TBM1			
Property	Unit	S1	S2	S3	Average
Shear modulus (G)	GPa	0.80	0.85	0.76	0.80±0.04
Maximum shear stress $\left(\tau_{u}\right)$	MPa	40.91	41.32	41.35	41.19±0.20
Shear strain at failure $\left(\gamma_{f}\right)$	mm/mm	0.1141	0.1303	0.1505	0.1316±0.01

**Table 16:** Shear properties of TTM1 adhesive.

		TTM1			
Property	Unit	S1	S2	S3	Average
Shear modulus (G)	GPa	0.81	0.72	0.67	0.73±0.06
Maximum shear stress $\left(\tau_{u}\right)$	MPa	38.49	36.14	36.11	36.91±1.11
Shear strain at failure $\left(\gamma_{f}\right)$	mm/mm	0.1167	0.1385	0.2171	0.1574±0.0431

**Table 17:** Shear properties of BBM2 adhesive.

		BBM2			
Property	Unit	S1	S2	S3	Average
Shear modulus (G)	GPa	1.21	1.27	1.31	1.26±0.04
Maximum shear stress $\left(\tau_{u}\right)$	MPa	49.29	52.43	53.23	51.65±1.70
Shear strain at failure $\left(\gamma_{f}\right)$	mm/mm	0.0513	0.0515	0.0588	0.0539±0.0035

**Table 18:** Shear properties of BTM2 adhesive.

		BTM2			
Property	Unit	S1	S2	S3	Average
Shear modulus (G)	GPa	2.26	2.06	2.12	2.15±0.08
Maximum shear stress $\left(\tau_{u}\right)$	MPa	46.33	46.28	47.02	46.54±0.34
Shear strain at failure $\left(\gamma_{f}\right)$	mm/mm	0.035	0.0395	0.0453	0.0399±0.0042

**Table 19:** Shear properties of TBM2 adhesive.

		TBM2			
Property	Unit	S1	S2	S3	Average
Shear modulus (G)	GPa	1.99	2.01	2.04	2.01±0.02
Maximum shear stress $\left(\tau_{u}\right)$	MPa	43.15	42.63	43.30	43.03±0.29
Shear strain at failure $\left(\gamma_{f}\right)$	mm/mm	0.0426	0.0426	0.0453	0.0435±0.0013

**Table 20:** Shear properties of TTM2 adhesive.

		TTM2					
Property	Unit	S1	S2	S3	S4	S5	Average
Shear modulus (G)	GPa	0.81	1.56	1.53	0.75	0.87	1.10±0.36
Maximum shear stress $\left(\tau_{u}\right)$	MPa	36.6	37.16	37.60	40.24	38.92	38.10±1.31
Shear strain at failure $\left(\gamma_{f}\right)$	mm/mm	0.1285	0.0824	0.1414	0.1654	0.1047	0.12±0.03

### 1.5 Single-edge-notch bending (SENB)

**Fig. 20:**
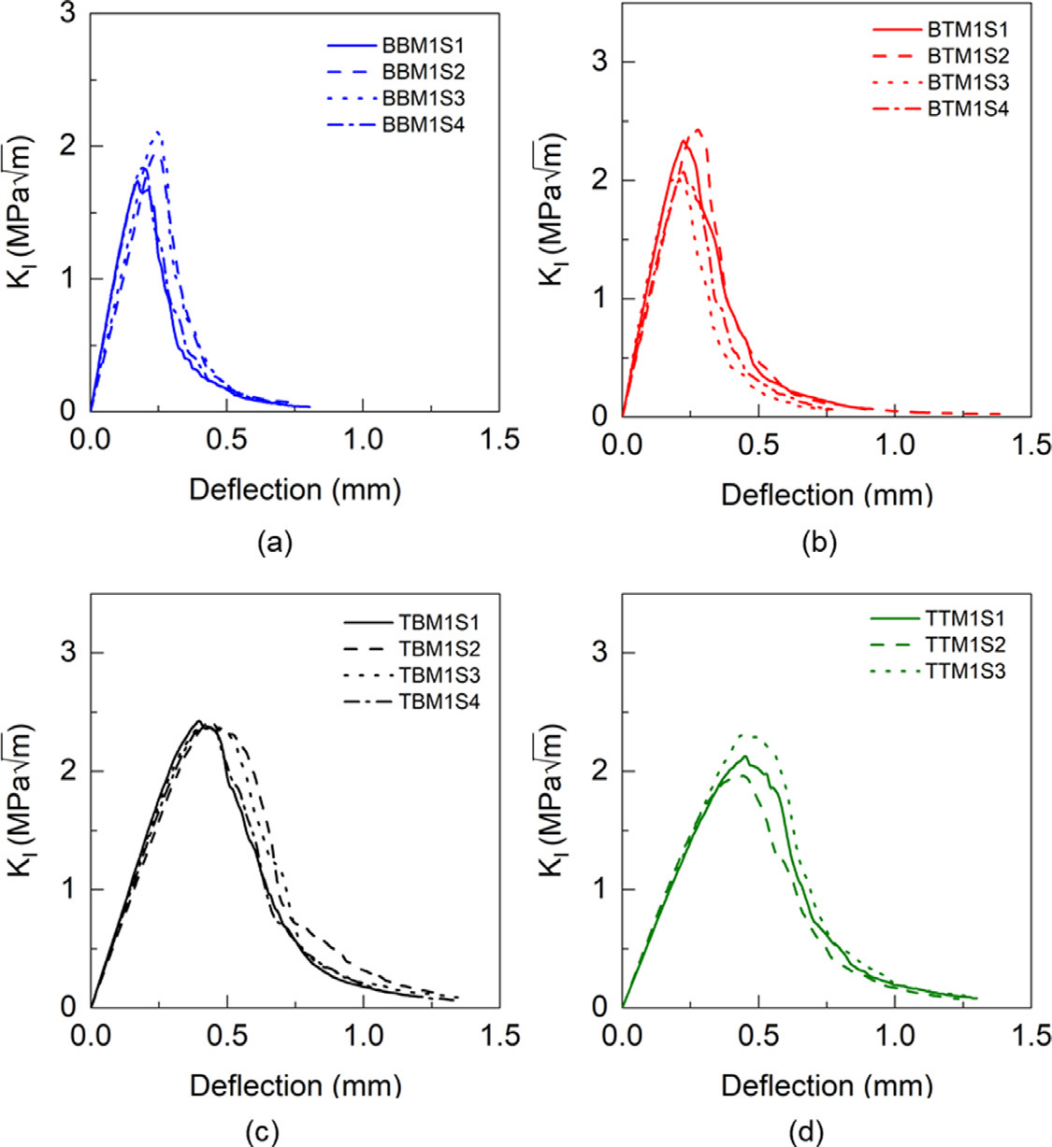
$K_{\mathrm{IC}}$ versus deflection response of BBM1 adhesive.

**Fig. 21:**
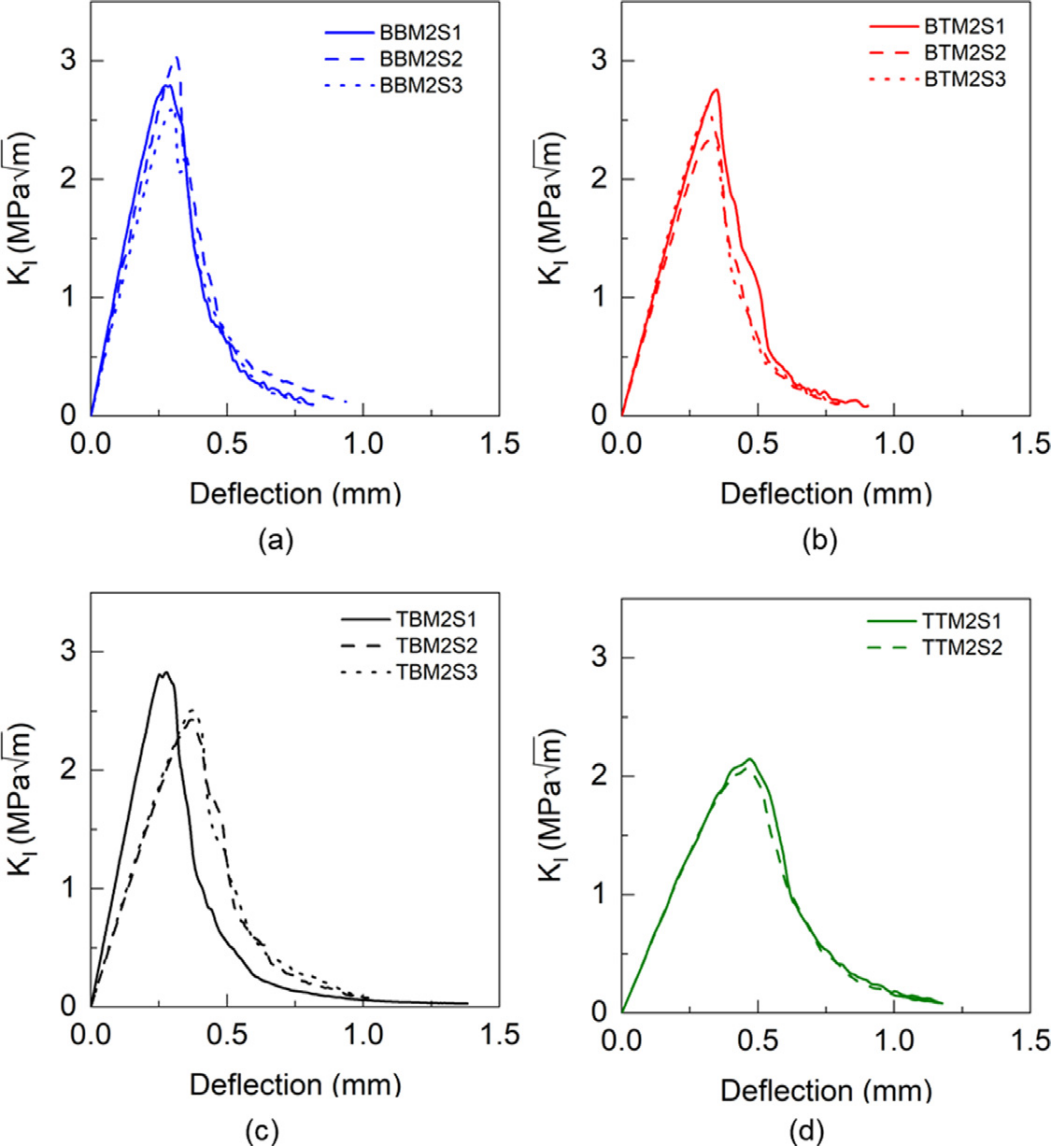
$K_{\mathrm{IC}}$ versus deflection response of BBM2 adhesive.

**Table 21:** Calculation of $K_{\mathrm{IC}}\;$of BBM1 adhesive.

		BBM1				
Property	Unit	S1	S2	S3	S4	Average
Initial crack length (a)	mm	4.2	4.32	4.47	4	
Thickness (t)	mm	4.64	4.66	4.64	4.65	
Load $\left(P_{Q}\right)$	N	62.54	66.92	67.42	71.77	
Fracture toughness $\left(K_{\text{IC}}\right)$	$\text{MPa}\sqrt{m}$	1.609	1.95	2.056	1.762	1.84±0.17

**Table 22:** Calculation of $K_{\mathrm{IC}}\;$of BTM1 adhesive.

		BTM1				
Property	Unit	S1	S2	S3	S4	Average
Initial crack length (a)	mm	4.6	4.6	4.2	4.55	
Thickness (t)	mm	4.92	5.02	5	5	
Load $\left(P_{Q}\right)$	N	67.58	78.82	76.36	65.9	
Fracture toughness $\left(K_{\text{IC}}\right)$	$\text{MPa}\sqrt{m}$	2.11	2.41	1.97	1.98	2.12±0.18

**Table 23:** Calculation of $K_{\mathrm{IC}}\;$of TBM1 adhesive.

		TBM1				
Property	Unit	S1	S2	S3	S4	Average
Initial crack length (a)	mm	4.33	4.45	4.57	4.25	
Thickness (t)	mm	4.2	4.15	4.13	4.21	
Load $\left(P_{Q}\right)$	N	67.32	64.47	58.39	67.2	
Fracture toughness $\left(K_{\text{IC}}\right)$	$\text{MPa}\sqrt{m}$	2.19	2.23	2.14	2.1	2.17±0.05

**Table 24:** Calculation of $K_{IC}\;$of TTM1 adhesive.

		TTM1			
Property	Unit	S1	S2	S3	Average
Initial crack length (a)	mm	4.26	4	4.5	
Thickness (t)	mm	4.96	4.9	4.9	
Load $\left(P_{Q}\right)$	N	60.96	64.54	69.7	
Fracture toughness $\left(K_{\text{IC}}\right)$	$\text{MPa}\sqrt{m}$	1.63	1.7	1.57	1.63±0.05

**Table 25:** Calculation of $K_{\mathrm{IC}}\;$of BBM2 adhesive.

		BBM2			
Property	Unit	S1	S2	S3	Average
Initial crack length (a)	mm	4.39	4.42	4.05	
Thickness (t)	mm	4	3.97	4.05	
Load $\left(P_{Q}\right)$	N	73.30	76.91	83.94	
Fracture toughness $\left(K_{\text{IC}}\right)$	$\text{MPa}\sqrt{m}$	2.68	2.77	2.48	2.64±0.12

**Table 26:** Calculation of $K_{\mathrm{IC}}\;$of BTM2 adhesive.

		BTM2			
Property	Unit	S1	S2	S3	Average
Initial crack length (a)	mm	4.37	4.41	4.09	
Thickness (t)	mm	3.9	3.89	3.9	
Load $\left(P_{Q}\right)$	N	72.63	59.86	76.38	
Fracture toughness $\left(K_{\text{IC}}\right)$	$\text{MPa}\sqrt{m}$	2.58	2.17	2.42	2.39±0.17

**Table 27:** Calculation of $K_{\mathrm{IC}}\;$of TBM2 adhesive.

		TBM2			
Property	Unit	S1	S2	S3	Average
Initial crack length (a)	mm	4.25	4.19	4.29	
Thickness (t)	mm	3.7	3.92	3.75	
Load $\left(P_{Q}\right)$	N	78.82	69.29	61.63	
Fracture toughness $\left(K_{\text{IC}}\right)$	$\text{MPa}\sqrt{m}$	2.81	2.27	2.2	2.43±0.27

**Table 28:** Calculation of $K_{IC}\;$of TTM2 adhesive.

		TTM2		
Property	Unit	S1	S2	Average
Initial crack length (a)	mm	4.14	4.3	
Thickness (t)	mm	3.86	3.88	
Load $\left(P_{Q}\right)$	N	60.09	59.16	
Fracture toughness $\left(K_{\text{IC}}\right)$	$\text{MPa}\sqrt{m}$	1.96	2.05	2.01±0.04

## 2 Experimental Design, Materials and Methods

### 2.1 Materials

Recent growth in wind energy requires new adhesive materials to accelerate the wind turbine blade production rate. Rapid curing non-toughened and toughened epoxy adhesives are typically used for bonding the wind turbine blade components. SPABOND™ 820HTA (non-toughened) and SPABOND™ 840HTA (toughened) adhesives were hybridized and manufactured by two different mixing techniques. The machine and manual mixing techniques are described as M1 and M2, respectively. The pristine and hybrid adhesive compositions are mentioned in Table 29. SP refers Spabond adhesive.

**Table 29:** Adhesive material composition.

Adhesive	Base	Hardener	Manufacturing method	Comments
BBM1	SP 820	SP 820	M1	
BTM1	SP 820	SP 840	M1	
TBM1	SP 840	SP 820	M1	
TTM1	SP 840	SP 840	M1	
BBM2	SP 820	SP 820	M2	
BTM2	SP 820 +SP 840	SP 820+ SP 840	M2	75:25 wt%
TBM2	SP 820+SP 840	SP 820+SP 840	M2	50:50 wt%
TTM2	SP 840	SP 840	M2	

### 2.2 Manufacturing

**Fig. 22:**
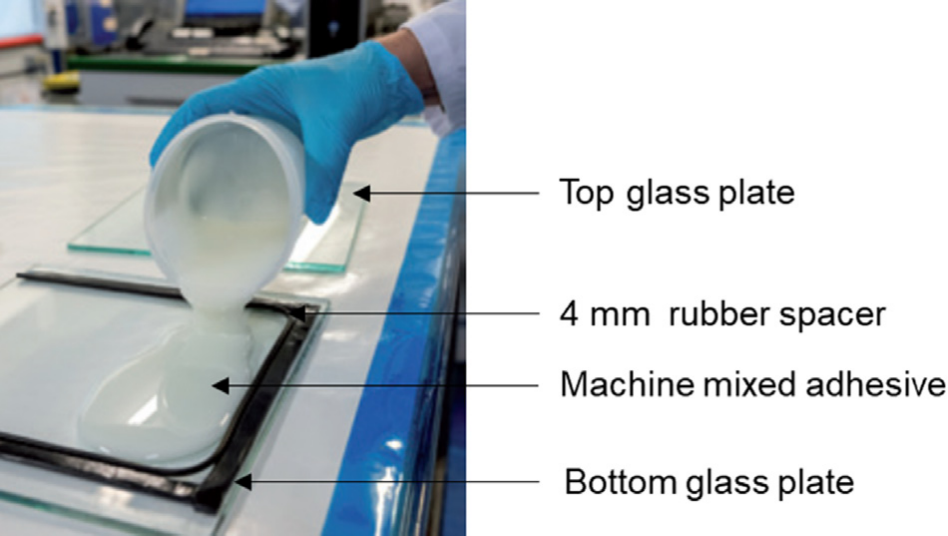
M1 manufacturing procedure [3].

The epoxy adhesive base and hardener material were weighed at a ratio of 100:33 and machine-mixed, as practiced by the wind turbine blade manufacturers. As shown in Fig. 22, the mixed adhesive was dispensed on the bottom glass plate. The top glass plate was used to distribute the adhesive uniformly. These M1 adhesive panels were fabricated and provided by Gurit (UK) Ltd.

**Fig. 23:**
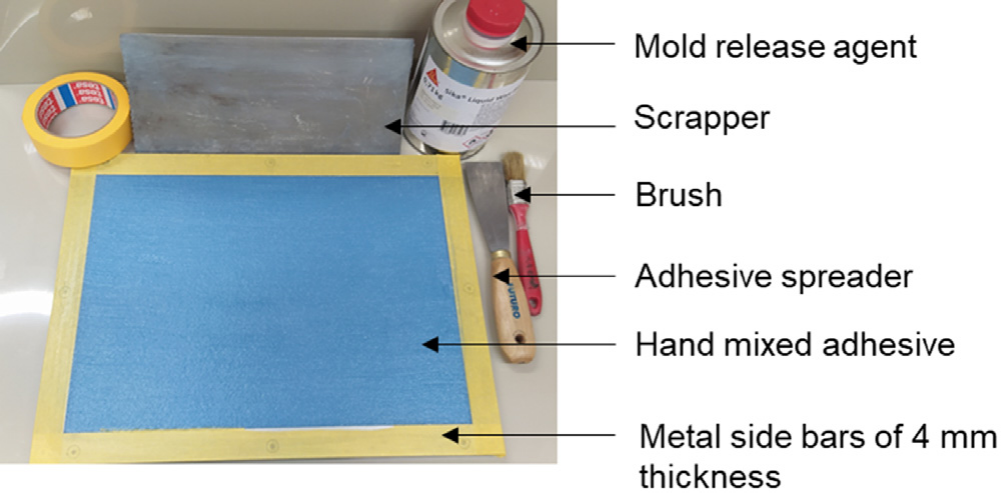
M2 manufacturing procedure.

The weighed adhesive base and hardener were manually mixed by using wooden spatula for 5–7 min . The M2, hand-mixing process introduced air bubbles that were partially removed by vacuum degassing for 7 min at a vacumm pressure of 0.95 bar. Due to the high viscous nature of adhesive, the air bubbles could not be eliminated. As depicted in Fig. 23, an aluminum plate having side bars of 4 mm thickness was coated twice with a mold release agent, Sika^Ⓡ^ liquid wax- 815 using a brush. The mold set up was dried for 15±5 min at the ambient temperature (20°C ± 2°C). The mixed adhesive was applied inside the mold cavity carefully using the adhesive spreader. Finally, the remaining adhesive were removed using a scraper.

**Fig. 24:**
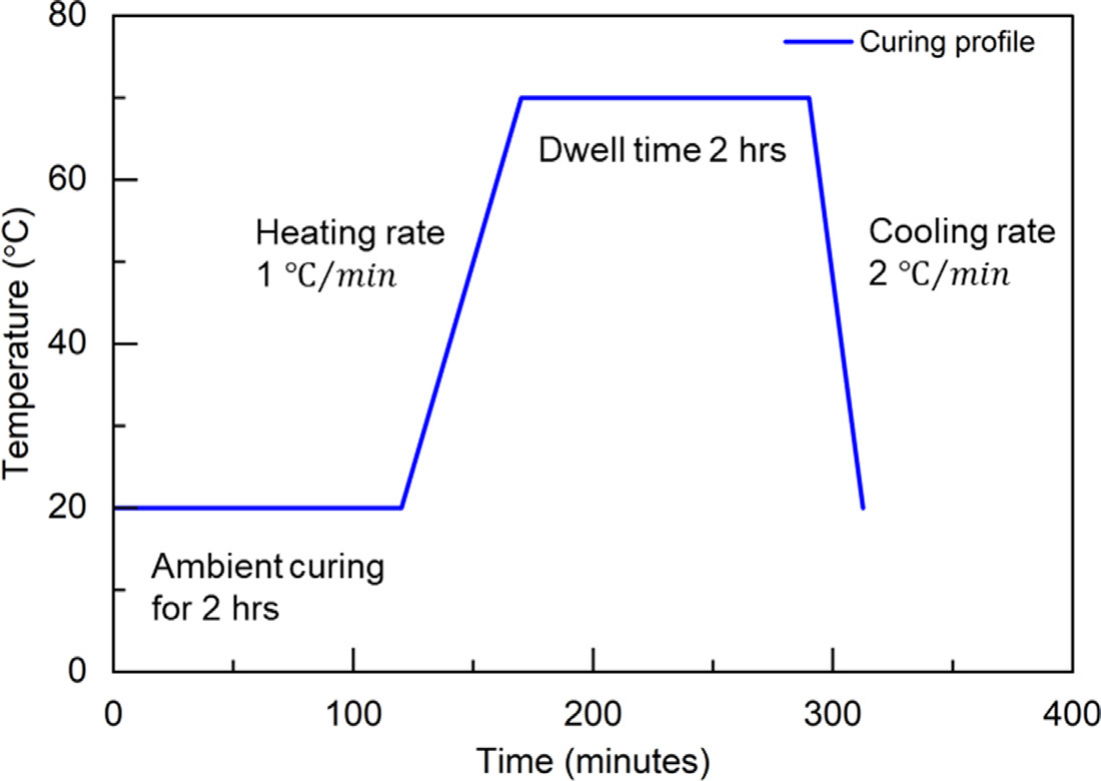
Curing profile of all adhesive panels.

Fig. 24 shows the curing cycle of the M1 and M2 adhesives. The ambient curing time includes the time for adhesive mixing and applying process. Thus, the time for bonding actual blades was taken into consideration. The curing profile was programmed and maintained by the Memmert^Ⓡ^ forced convection oven.

Following the curing procedure, the DMA, tensile, V-notch and SENB test specimens were cut to the required dimensions using wate-jet cutting machine.

### 2.3 Experimental methods

**Fig. 25:**
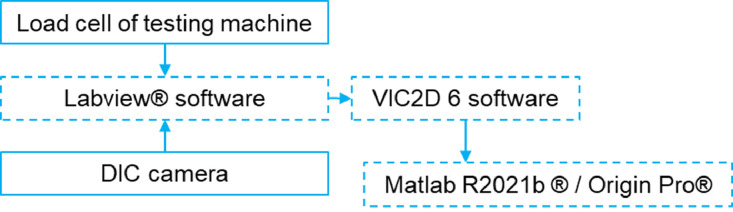
DIC data collection in tensile, V-notch shear and SENB testing.

The experimental set up details of micro CT scan, DMA, tensile, V-notch and SENB experiments are provided in Table 30, Table 31, Table 32, Table 33, Table 34 and 34, respectively. The force values from the test machine and the corresponding images from camera were collected by the Labview^Ⓡ^ software (Fig. 25). The same frequency of 0.5 Hz (SENB) or 1 Hz (tensile and V-notch shear) used for the data acquisition. Further, the images were analysed by VIC2D 6 software to determine the engineering strain (tensile and V-notch shear) and deflection (SENB). The strain/delection values were matched with the recorded load values. Further the material properties were analysed with Matlab R2021b^Ⓡ^ software.

**Table 30:** Micro CT experimental setup and parameters.

Experimental setup	Comment
Nominal dimension	15 mm × 13 mm × 4 mm
Test equipment	Ultratom µCT system
Source-detector distance	620 mm
Voxel size	3.5 µm
Number of projections	1632
Step size	0.22°
Exposure time	0.25 s

**Table 31:** DMA experimental setup and parameters.

Experimental setup	Comment
Nominal dimension	35 mm × 10 mm × 4 mm
Test equipment	TA^Ⓡ^ Q800 series
Mode	Single cantilever mode
Testing amplitude	20 µ*m*
Testing frequency	1 Hz
Testing temperature range	-50°C to 150°C
Heating rate	5${\;}^{\circ }C/min$.
ASTM standard	ASTM D7028-07 (2015)
Measured quantities	Storage modulus and tan δ
Software	TA universal analysis^Ⓡ^

**Table 32:** Uniaxial tensile experimental setup and parameters.

Experimental setup	Comment
Nominal dimension	Refer Type I, dog-bone specimen
Test equipment	MTS^Ⓡ^ 810 Landmark servo-hydraulic machine
Load cell capacity	Calibrated for 5 kN with accuracy of ±0.2%.
Displacement rate	1 mm/min
Strain measurement	DIC technique
Camera	Point Grey – Grasshopper 3 camera (2.2 Megapixels) housing Fujinon HF35SA-1 35 mm F/1.4 lens.
DIC analysis software	VIC2D 6
ASTM standard	ASTM D638-14
DAQ software	Labview^Ⓡ^

**Table 33:** V-notch shear experimental setup and parameters.

Experimental setup	Comment
Nominal dimension	76 mm × 19 mm × 4 mm
Test equipment	Walter + bai (w + b)
Load cell capacity	Calibrated for 50 kN with accuracy of ±0.2%.
Displacement rate	1 mm/min
Strain measurement	DIC technique
Camera	Sony XCG-5005E (5 Megapixels)
DIC analysis software	VIC2D 6
ASTM standard	ASTM D5379-19
DAQ software	Labview^Ⓡ^

**Table 34:** SENB experimental setup and parameters.

Experimental setup	Comment
Nominal dimension	35.2 mm × 8 mm × 4 mm
Test equipment	MTS^Ⓡ^ Acumen
Load cell capacity	3 kN
Displacement rate	0.25 mm/min
Deflection measurement	DIC technique
DIC analysis software	VIC2D 6
Camera	Sony XCG-5005E (5 Megapixels)
ASTM standard	ASTM D5379-19
DAQ software	Labview^Ⓡ^

## Ethics Statement

This work did not involve human subjects, animal experiments, or data collected from social media platforms.

## CRediT authorship contribution statement

**Dharun Vadugappatty Srinivasan:** Conceptualization, Methodology, Data curation, Writing – original draft, Investigation. **Anastasios P. Vassilopoulos:** Conceptualization, Supervision, Validation, Writing – review & editing, Funding acquisition.

## Declaration of Competing Interest

The authors declare that they have no known competing financial interests or personal relationships that could have appeared to influence the work reported in this paper.

## Data Availability

Hybrid epoxy adhesive properties for wind turbine blade applications (Original data) (Mendeley Data).

## References

[1] Srinivasan D.V., Vassilopoulos A.P. (2022). Manufacturing and toughening effects on the material properties of wind turbine blade adhesives. Polym. Test..

[2] D. V. Srinivasan, A.P. Vassilopoulos, Hybrid epoxy adhesive properties for wind turbine blade applications, 1 (2022). 10.17632/5RGWZW6JN3.1 .

[3] SHAPE - the Gurit magazine #19, (2020). https://issuu.com/gurit/docs/gurit_shape-magazine_19-2020_en (accessed July 2, 2022)

